# Tryptase Regulates the Epigenetic Modification of Core Histones in Mast Cell Leukemia Cells

**DOI:** 10.3389/fimmu.2021.804408

**Published:** 2021-12-02

**Authors:** Sultan Alanazi, Fabio Rabelo Melo, Gunnar Pejler

**Affiliations:** Department of Medical Biochemistry and Microbiology, Uppsala University, Uppsala, Sweden

**Keywords:** tryptase, histones, mast cells, epigenetics, apoptosis

## Abstract

Mast cells are immune cells that store large amounts of mast cell-restricted proteases in their secretory granules, including tryptase, chymase and carboxypeptidase A3. In mouse mast cells, it has been shown that tryptase, in addition to its canonical location in secretory granules, can be found in the nuclear compartment where it can impact on core histones. Here we asked whether tryptase can execute core histone processing in human mast cell leukemia cells, and whether tryptase thereby can affect the epigenetic modification of core histones. Our findings reveal that triggering of cell death in HMC-1 mast cell leukemia cells is associated with extensive cleavage of core histone 3 (H3) and more restricted cleavage of H2B. Tryptase inhibition caused a complete blockade of such processing. Our data also show that HMC-1 cell death was associated with a major reduction of several epigenetic histone marks, including H3 lysine-4-mono-methylation (H3K4me1), H3K9me2, H3 serine-10-phosphorylation (H3S10p) and H2B lysine-16-acetylation (H2BK16ac), and that tryptase inhibition reverses the effect of cell death on these epigenetic marks. Further, we show that tryptase is present in the nucleus of both viable and dying mast cell leukemia cells. In line with a role for tryptase in regulating nuclear events, tryptase inhibition caused increased proliferation of the mast cell leukemia cells. Altogether, the present study emphasizes a novel principle for how epigenetic modification of core histones is regulated, and provides novel insight into the biological function of human mast cell tryptase.

## Introduction

Mast cells are hematopoietic cells of the immune system. They are implicated in various pathological conditions, including allergic conditions, autoimmune disorders, cancer and cardiovascular diseases (1–3). In addition, mastocytosis is a pathological condition in which the mast cell population is expanded. Typically, the latter occurs through a D816E mutation in KIT, i.e., the receptor for stem cell factor. This mutation results in constitutive KIT activation, and since stem cell factor is a critical growth factor for mast cells, constant KIT signaling will result in excessive mast cell proliferation (4). Mastocytosis is a rare disorder and is subclassified into cutaneous and systemic forms, with the systemic form being further subclassified into indolent and advanced variants, the latter encompassing aggressive systemic mastocytosis and mast cell leukemia (4).

A hallmark feature of mast cells is their high content of secretory granules. These contain vast amounts of preformed bioactive inflammatory mediators, including various amines (e.g., histamine, serotonin), proteoglycans of serglycin type, lysosomal hydrolases, cytokines (e.g., TNF), growth factors (e.g., VEGF), and a panel of mast cell-restricted proteases (5). The latter category encompasses tryptase, chymase and carboxypeptidase A3 (CPA3), and these proteases are stored in remarkably high amounts (5, 6). The mast cell proteases have been shown to account for the detrimental impact of mast cells in various pathological settings (5–9), but they also have beneficial functions, in particular in host defense against various toxins (10–12).

Among the mast cell proteases, tryptase has a unique macromolecular organization, being built up as a tetrameric enzyme with all of its active sites facing a central, narrow pore (13). Due to this organization, tryptase is completely resistant to the action of endogenous, macromolecular protease inhibitors. Moreover, since most macromolecular substrates are not able to enter the central pore of tryptase, the enzyme has a relatively narrow substrate cleavage repertoire (13, 14).

In a previous study, we noted that tryptase expressed by mouse primary mast cells has the capacity to execute N-terminal cleavage of nuclear core histones in mast cells undergoing apoptotic cell death (15). We also demonstrated that tryptase, in addition to its canonical location within the mast cell secretory granules, also can be found in the nucleus of primary, cultured mouse mast cells (15). It is now well established that the N-terminal portions of nuclear core histones are important sites for deposition of epigenetic marks, including acetylation, phosphorylation and methylation (16, 17). N-terminal cleavage of core histones by tryptase could potentially serve to remove such epigenetic histone marks. Hence, any biological effects mediated by the respective marks will be erased though the action of tryptase. In support of this notion, we found previously that murine mast cells lacking tryptase expression display an elevation in the levels of histone 2B (H2B) lysine-5 acetylation (H2BK5ac) (18).

Although an effect of tryptase on core histones has been detected in primary mouse mast cells, the possibility that tryptase has an effect on the processing and epigenetic modification of core histones in human mast cells has not been explored. In this study we addressed this possibility by examining the effects of tryptase on core histone processing and epigenetic modification in human mast cell leukemia cells undergoing cell death. Moreover, we investigated whether tryptase can be found in the nucleus of mast cell leukemia cells and if tryptase can have an impact on mast cell leukemia cell proliferation.

## Results

### H-Leu-Leu-OMe (LLME), Staurosporine and Histone Methyltransferase Inhibitors Are Cytotoxic for HMC-1 Mast Cells

To investigate the impact of tryptase on histone integrity during mast cell death, we first evaluated cell death responses of HMC-1 cells to various cytotoxic agents. To this end, HMC-1 cells were treated with either H-Leu-Leu-OMe (LLME) [a lysosomotropic agent with mast cell granule-permeabilizing properties (19)], staurosporine (an agent known to cause caspase-dependent apoptosis), UNC-0638 or UNC-1999; the latter compounds represent histone methyltransferase inhibitors with known ability to induce cell death in transformed mast cells (20). These analyses revealed that all of the employed agents were cytotoxic to the HMC-1 cells (**Supplementary Figure 1A**). A further analysis showed that all agents caused cell death with signs of both apoptosis (Annexin V^+^/Draq7^-^) and necrosis/late apoptosis (Annexin V^+^/Draq7^+^) (**Supplementary Figure 1B, C**). Notably, apoptotic cell death dominated in response to LLME, in agreement with previous findings in which LLME was shown to cause apoptosis in primary mouse mast cells (19, 21).

### LLME, Staurosporine and Histone Methyltransferase Inhibitors Cause Histone 3 Processing in HMC-1 Mast Cells

**Figure 1:**
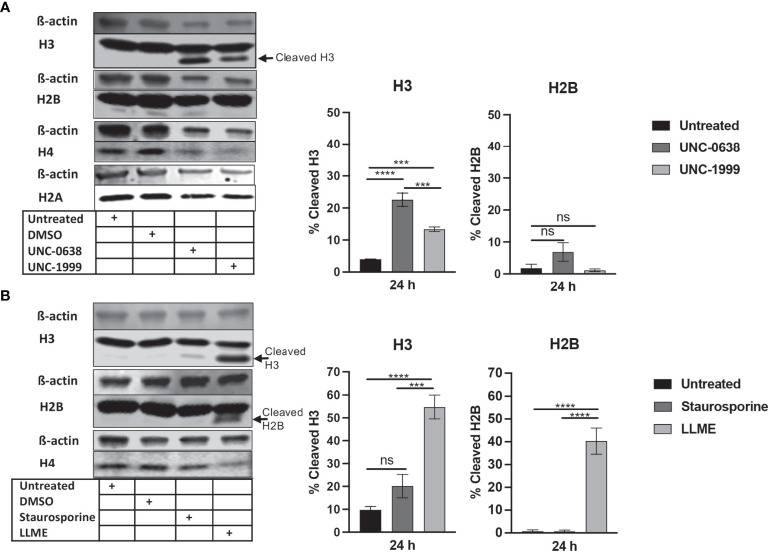
Histone 3 (H3) is cleaved in human mast cell leukemia cells in response to cytotoxic agents. HMC-1 cells (0.5 x 10^6^ cells/ml) were either left untreated or incubated for 24 h with either **(A)** UNC-0638 (25 μM), UNC-1999 (50 μM), **(B)** staurosporine (1 μM) or H-Leu-Leu-OMe (LLME) (400 μM). Samples corresponding to equal numbers of cells were subjected to Western blot analysis for histone 3 (H3), H2B, H4 and H2A, with ß-actin as loading control. Quantification of the signal intensity for cleaved H3 and H2B, as % of total H3 and H2B, is shown in the right part of the panels. The presented data are representative of at least two independent experiments and are given as mean values ± SEM (n=3). ***p ≤ 0.001; ****p ≤ 0.0001. ns, not significant.

To investigate the impact of cell death on core histone processing in the HMC-1 cells, we performed Western blot analysis. As shown in **Figure 1A**, the intensities of the bands corresponding to the respective core histones (H3, H2B, H4, H2A) were generally reduced in response to UNC-0638 or UNC-1999 (**Figure 1A**), most likely reflecting a general decline in cellular protein levels during the cell death process; this was supported by a general reduction in β-actin levels (**Figure 1A**). Interestingly, limited cleavage of H3 was seen in response to UNC-0638, and also to some extent in response to UNC-1999 (**Figure 1A**; quantification of band intensities is shown in the right part of the panel), suggesting that histone processing may accompany cell death in response to these agents. Cleavage of H2B, H2A or H4 was not observed. Limited H3 cleavage was also seen in response to staurosporine (**Figure 1B**), and profound H3 cleavage was seen in response to LLME (**Figure 1B**); LLME also caused cleavage of H2B. Altogether, these findings indicate that cell death of HMC-1 cells, in response to various cytotoxic agents, is accompanied by histone cleavage. Moreover, out of the assessed cytotoxic agents, LLME had the most extensive effects on core histone cleavage in the HMC-1 cells.

### Cell Death in HMC-1 Cells Is Associated With a Reduction in the H3K4me1, H3K9me2 and H3K27me3 Epigenetic Marks

**Figure 2:**
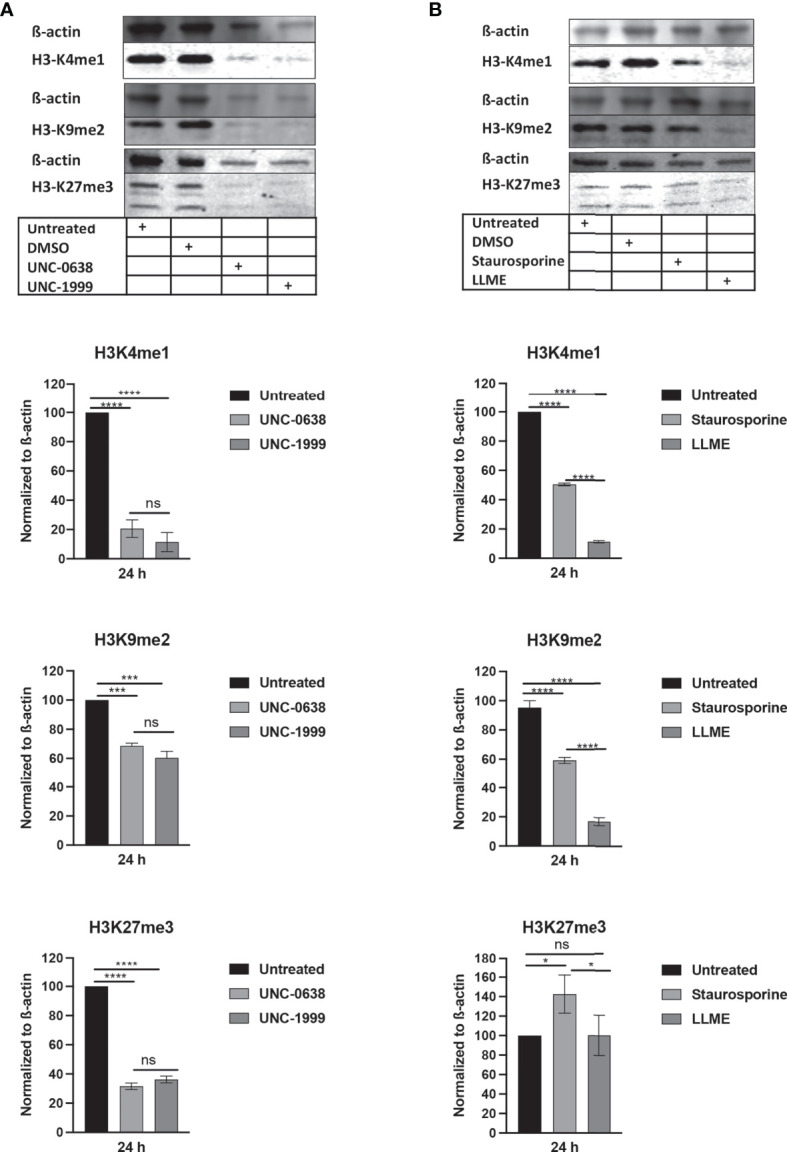
Cytotoxic agents cause reductions in the levels of epigenetic histone marks in mast cell leukemia cells. HMC-1 cells (0.5 × 10^6^ cells/ml) were incubated for 24 hours with **(A)** UNC-0638 (25 μM), UNC-1999 (50 μM), **(B)** staurosporine (1 μM) or H-Leu-Le (400 μM), followed by Western blot analysis for the levels of the H3K4me1, H3K9me2 and H3K27me3 marks, with ß-actin as loading control. Quantification of H3K4me1, H3K9me2 and H3K27me3 levels by densitometry is shown in the lower part of the panels. Data are representative of three independent experiments and are given as mean values ± SEM (n=3). *p ≤ 0.05; ***p ≤ 0.001; ****p ≤ 0.0001. ns, not significant.

Next, we assessed whether cell death in HMC-1 cells is accompanied by effects on the levels of epigenetic histone marks, based on the notion that apoptosis is associated with extensive histone modifications (22). For this purpose, we examined the effects of LLME, staurosporine, UNC-0638 or UNC-1999 on the levels of the H3 lysine-4 mono-methylation (H3K4me1), H3 lysine-9 di-methylation (H3K9me2) and H3 lysine-27 tri-methylation (H3K27me3) epigenetic marks. As shown in **Figure 2A**, a robust reduction of H3K4me1 and H3K27me3, as well as a minor rection of H3K9me2, was seen in response to both UNC-0638 and UNC-1999. Moreover, a profound reduction in the levels of H3K4me1 and H3K9me2 was seen in response to LLME (**Figure 2B**). A more limited reduction of H3K4me1 and H3K9me2 was seen in response to staurosporine, whereas neither LLME nor staurosporine caused a reduction of H3K27me3 levels.

### Tryptase Inhibition Causes Increased Late Apoptosis/Necrosis Over Apoptosis in HMC-1 Cells

The findings above reveal that cell death in HMC-1 cells is accompanied by extensive core histone processing and alterations in the levels of epigenetic histone marks. Considering that tryptase has previously been shown to regulate such processes in primary murine mast cells (15, 18), we next considered the possibility that the effects on core histone processing/epigenetic modification in HMC-1 cells could be dependent on tryptase. To approach this issue, we first assessed whether the inhibition of tryptase, by using either a general seine protease inhibitor (Pefabloc SC) or a potent tryptase inhibitor (nafamostat) (23), could have an impact on the cell death process in HMC-1 cells. Moreover, since we noted above that cell death-induced core histone cleavage and epigenetic modification was most profound in response to LLME, we focused in the following on LLME out of the assessed cytotoxic agents.

**Figure 3:**
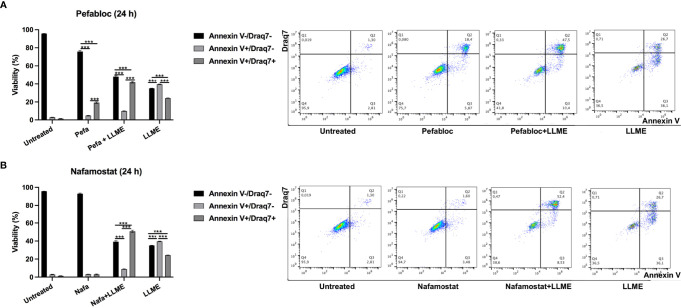
Tryptase inhibition alters the mode of cell death in human mast cell leukemia cells subjected to cytotoxic agent. HMC-1 cells (0.5 x 10^6^ cells/ml) were incubated with either Pefabloc SC (**A**; 100 μM) or nafamostat (**B**; 20 μM) for 30 min prior to treating the cells with LLME (400 μM) for 24 h. Cells were analyzed by flow cytometry for Annexin V/Draq7 positivity. Dot plots are shown with representative samples showing staining with Annexin V-FITC (FL-1) and Draq7 (FL-3), with the % of cells indicated within each quadrant. Quantification of the data is shown to the left. Data are representative of at least two independent experiments and are given as mean values ± SEM (n = 3). ***p ≤ 0.001.

As seen in **Figures 3A, B** and **Supplementary Figure 1**, LLME caused predominantly apoptotic cell death in HMC-1 cells. However, in the presence of either Pefabloc SC (**Figure 3A**) or nafamostat (**Figure 3B**), a profound reduction in the proportion of apoptotic (Annnexin V^+^/Draq7^-^) cells was seen, and this was accompanied by a corresponding increase in the population of late apoptotic/necrotic (Annexin V^+^/Draq7^+^) cells. Nafamostat was not cytotoxic to the HMC-1 cells (**Figure 3B**), whereas limited cytotoxicity of Pefabloc SC was seen (**Figure 3A**). Hence, these findings reveal that tryptase impacts on the mechanism of cell death of HMC-1 cells in response to LLME.

### Tryptase Inhibition Blocks H3 Cleavage in HMC-1 Cells

**Figure 4:**
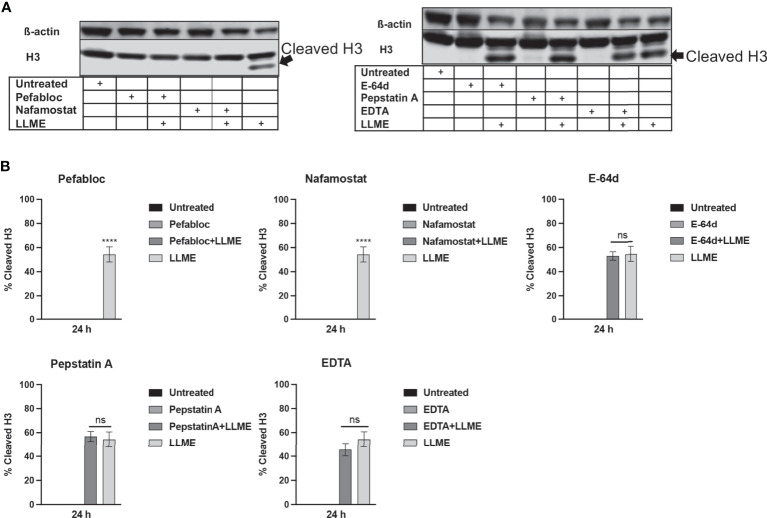
Treatment of mast cell leukemia cells with tryptase inhibitor abrogates H3 cleavage in response to cytotoxic agents. **(A)** HMC-1 cells (0.5 x 10^6^ cells/ml) were incubated with protease inhibitors: Pefabloc SC (Pefa; 0.1 mM), nafamostat (Nafa; 20 μM), E-64d (15 μM), Pepstatin A (Pep A; 50 μM) or EDTA (20 μM) for 30 min prior to treating the cells with LLME (400 μM) for 24 h. Subsequently, samples corresponding to equal numbers of cells were subjected to Western blot analysis for histone 3 (H3), with ß-actin as loading control. **(B)** Quantification of the signal intensity for cleaved H3, as % of total H3. The presented data are representative of at least two independent experiments, and are given as mean values ± SEM (n=3). ****p ≤ 0.0001. ns, not significant.

Next, we sought to investigate whether the LLME-induced cleavage of H3 was dependent on tryptase. As seen in **Figures 4A, B**, extensive cleavage of H3 was seen in response to LLME, confirming our findings above. However, when cells had been treated with either Pefabloc SC or nafamostat, the H3 processing was completely abolished. In contrast, the H3 cleavage was not affected in the presence of general inhibitors of cysteine proteases (E-64d), aspartic acid proteases (Pepstatin A) or metalloproteases (EDTA). Hence, these findings indicate that tryptase has a major role in executing H3 cleavage in the context of cell death in HMC-1 cells.

### Tryptase Inhibition Modulates Epigenetic Histone Modification in HMC-1 Cells

**Figure 5:**
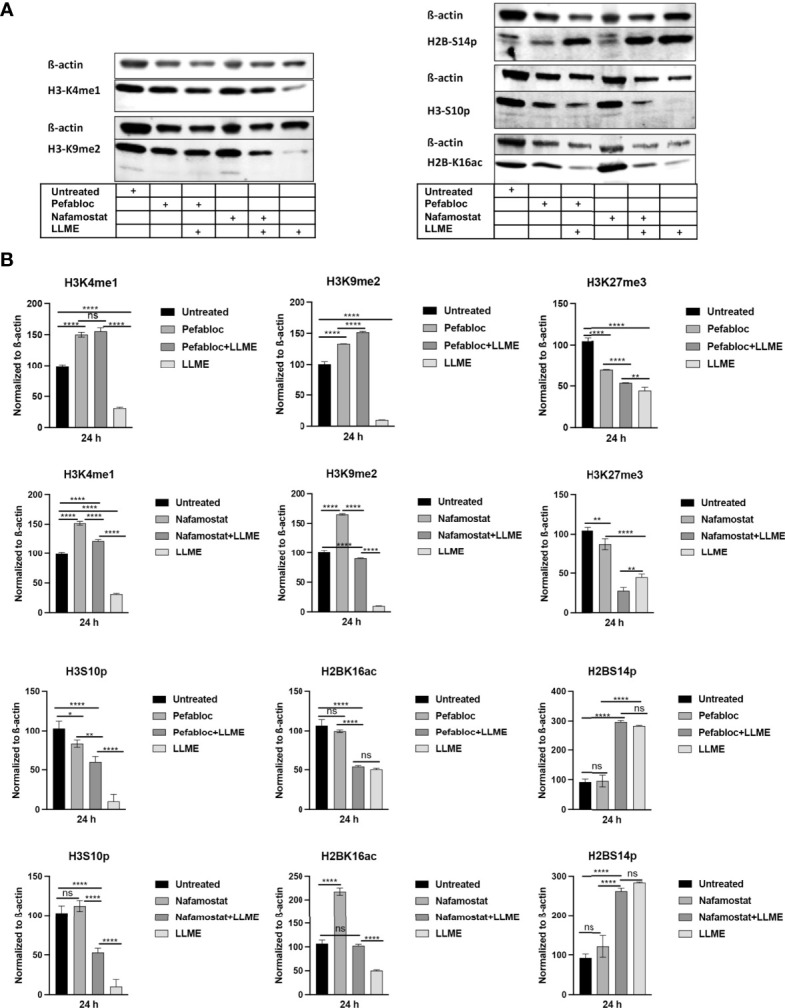
Tryptase inhibition blocks the effect of cytotoxic agents on the levels of epigenetic histone marks in mast cell leukemia cells. **(A)** HMC-1 cells (0.5 x 10^6^ cells/ml) were either left untreated or treated with either Pefabloc SC (Pefa; general serine protease inhibitor) or nafamostat (Nafa; tryptase inhibitor) for 30 min prior to incubation with LLME (400 μM) for 24 h. Samples corresponding to equal numbers of cells were subjected to Western blot analysis for levels of the H3K4me1, H3K9me2, H3K27me3, H2BS14p, H3S10p and H2BK16ac marks, with ß-actin used as loading control. **(B)** Quantification of the levels of the H3K4me1, H3K9me2, H3K27me3, H2BS14p, H3S10p, and H2BK16ac marks, normalized to the loading control. The presented data are representative of at least two independent experiments and are given as mean values ± SEM (n=3). *p ≤ 0.05; **p ≤ 0.01; ****p ≤ 0.0001. ns, not significant.

Extending from the observation that tryptase causes H3 cleavage in HMC-1 cells during cell death, we then proceeded to investigate whether tryptase thereby could modify the pattern of epigenetic H3 marks in the HMC-1 cells. First, we investigated the impact of tryptase inhibition on the levels of the H3K4me1 and H3K9me2 marks, considering that the levels of both of these are affected during LLME-induced cell death (see **Figure 2**). As seen in **Figures 5A, B**, tryptase inhibition by either Pefabloc SC or nafamostat completely reversed the effect of LLME on the levels of both these marks. Moreover, both Pefabloc SC and nafamostat caused a slight increase in the levels of the H3K4me1 and H3K9me2 marks also at baseline conditions, i.e., in the absence of cell death-inducing agent (**Figures 5A, B**).

To extend these findings, we also analyzed whether tryptase inhibition could impact on the levels of the H2B serine-14 phosphorylation (H2BS14p), H3 serine-10 phosphorylation (H3S10p) and H2B lysine-16 acetylation (H2BK16ac) marks. Out of these, the H2BS14p mark has a documented role in apoptosis, typically being upregulated in response to cell death (24–26). The H3S10p mark has an important role in chromatin remodeling during the mitotic process (27) but is also implicated in apoptosis (28). Further, the H2BK16ac mark has been shown to be involved in regulation of the cell cycle (29). As depicted in **Figures 5A, B**, cell death induced by LLME caused a robust reduction in the levels of the H3S10p mark, and it was also seen that tryptase inhibition by either Pefabloc SC or nafamostat attenuated the effect of LLME on H3S10p levels. LLME also caused a reduction in the levels of the H2BK16ac mark, and this effect was blunted in the presence of nafamostat, whereas Pefabloc SC was not capable of reverting the effects of LLME on H2BK16ac levels. Further, cell death in response to LLME was accompanied by a robust increase in the levels of the H2BS14p mark. However, neither nafamostat nor Pefabloc SC had the capacity to reverse the effect of LLME on the levels of this epigenetic mark.

### Tryptase Is Found in the Nucleus of HMC-1 Cells

**Figure 6:**
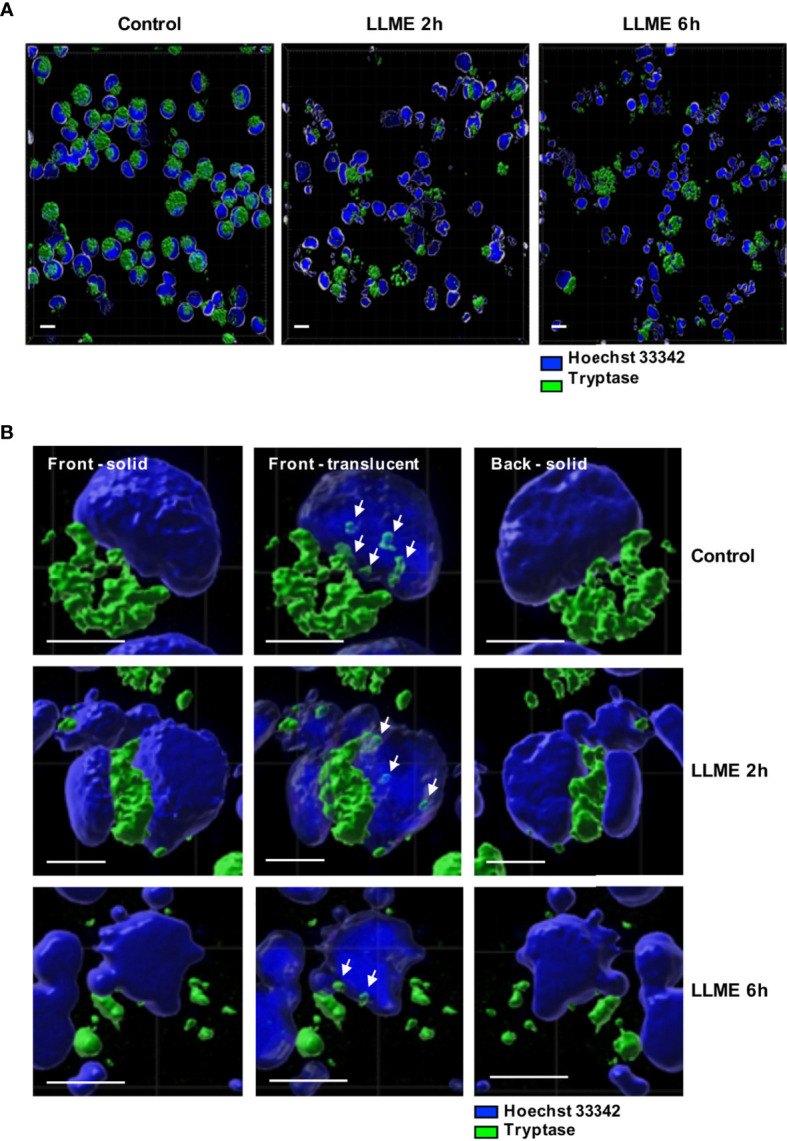
Tryptase is found in the nucleus of HMC-1 cells. HMC-1 cells (0.5 x 10^6^ cell/ml) were either left untreated or treated with 400 μM LLME for 2 or 6 hours. Cells were stained for tryptase and nuclear DNA (Hoechst 33342), followed by confocal microscopy analysis. **(A)** 3-D view generated from Z-stack sections. Note the abundant tryptase staining in the cytoplasm of control cells and a gradual decline in tryptase staining after LLME treatment; bar scale = 10 μm. **(B)** The left and right panels show solid front and back views of the same control cell or cells treated with LLME for either 2 or 6 hours. The middle panels show translucent nuclear structure. Note that the translucent depiction of nuclei reveals the presence of tryptase within the nuclear compartment of both control- and LLME-treated cells (indicated by white arrows); bar scale = 5 μm.

The observed impact of tryptase on nuclear histones implies that tryptase is physically associated with these proteins, either at baseline conditions or as a consequence of the apoptotic process. To address this, we stained the HMC-1 cells for tryptase, both at baseline conditions and after treatment with LLME. As seen in **Figures 6A, B**, HMC-1 cells showed strong cytoplasmic staining for tryptase, in agreement with the large quantities of tryptase typically found in the secretory granules. Tryptase positivity was also seen after LLME-induced cell death. However, a gradual decline in tryptase staining was seen after induction of cell death, most likely reflecting the release or degradation of tryptase during the cell death process. In accordance with previous finding in mouse mast cells (15), tryptase staining was also seen in the nuclear compartment. This was observed both at baseline conditions and after induction of cell death by LLME. As expected, LLME treatment caused extensive disintegration of the nuclear compartment of the HMC-1 cells, and it was also seen that the levels and distribution of nuclear tryptase was relatively similar at baseline vs. cell death conditions.

### Tryptase Inhibition Causes Increased HMC-1 Cell Proliferation

**Figure 7:**
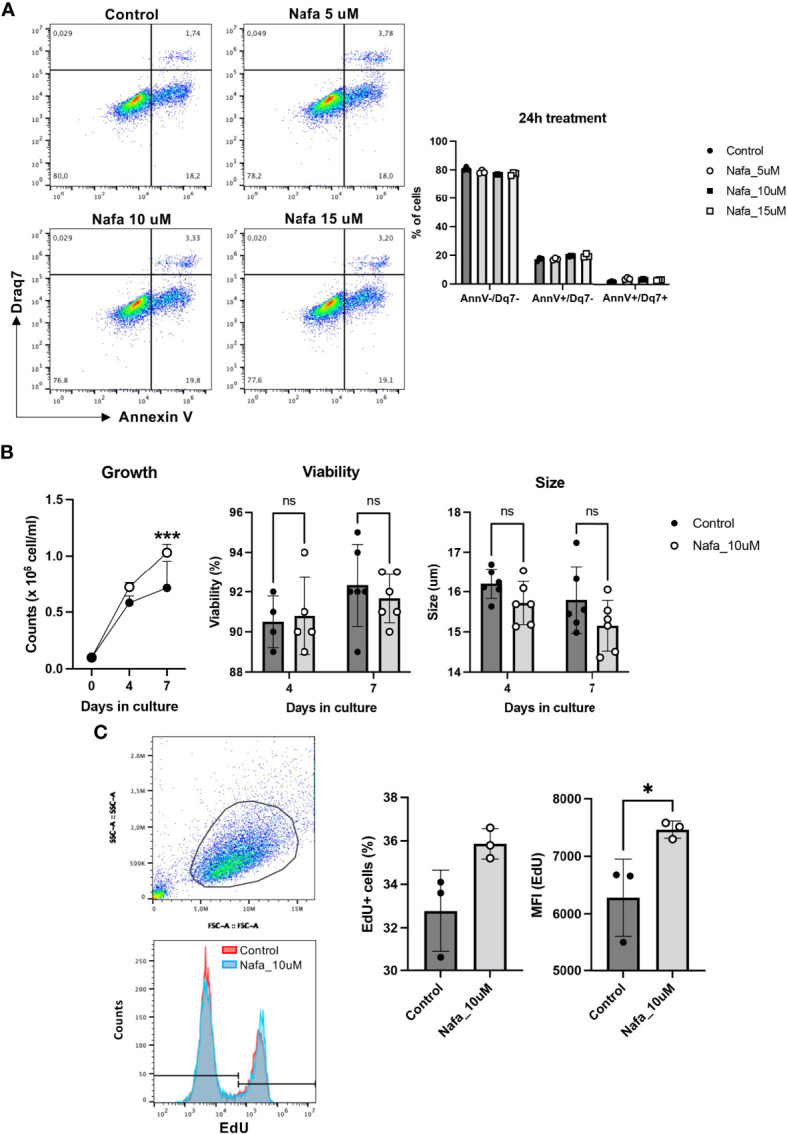
Nafamostat increases proliferation of HMC-1 cells. HMC-1 (0.1 x 10^6^ cell/ml) were left untreated or cultured in presence of nafamostat. **(A)** Flow cytometry analysis showing representative dot plots and cell viability of HMC-1 cells after 24h treatment with different concentrations of nafamostat; quantification is shown in the right panel. **(B)** Cell counts in control cultures and in cultures containing 10 μM nafamostat. Note the increase in cell numbers when cells are cultured in the presence of nafamostat. No effect of nafamostat was seen on cell viability as measured by trypan-blue staining or on cell size. **(C)** Cells were assessed for proliferation by EdU staining and flow cytometry analysis. Representative dot blot shows gated alive cells (upper left panel) and merged representative histograms for the EdU staining (left lower panel). Quantification of the EdU staining as % EdU^+^ cells and mean fluorescence intensity (MFI) is depicted in the right panel. Data are represented as mean values ± SEM and analyzed with 2-way ANOVA and unpaired *t* test. *p ≤ 0.05, ***p ≤ 0.0001. ns, not significant.

Our findings above reveal that tryptase has an impact on events occurring during cell death, and our data also demonstrate that tryptase is found in the HMC-1 cell nucleus, both at baseline conditions and in the context of cell death. Intriguingly, our findings also suggest that tryptase has an impact on the levels of certain epigenetic marks at baseline conditions. Altogether, this introduces the possibility that tryptase might impact on nuclear events even at baseline conditions, a notion that is supported by previous studies showing that the knockout of tryptase in murine primary mast cells results in loss of proliferative control (18). To assess whether tryptase could have an impact on proliferation also in transformed, human mast cells, we next investigated whether tryptase inhibition has an effect on the ability of HMC-1 cells to proliferate. For this purpose, HMC-1 cells were cultured in the absence or presence of nafamostat, followed by assessment of cell death (Annexin V/Draq7 staining) and quantification of cell numbers. This analysis revealed that nafamostat was not cytotoxic to the HMC-1 cells (**Figure 7A**), and also revealed a more rapid increase in cell numbers when cells were cultured in the presence of the tryptase inhibitor (**Figure 7B**). To assess whether the increased growth rate was due to increased proliferation, we adopted EdU staining. Indeed, this analysis revealed a markedly higher portion of proliferating cells in the presence of nafamostat (**Figure 7C**), suggesting that tryptase inhibition has a stimulatory impact on HMC-1 cell proliferation.

## Discussion

Tryptase is one of the established biomarkers for mastocytosis, with an increase in tryptase levels being one out several criteria for diagnosing a patient with mastocytosis (30). Tryptase is also a well-established biomarker for anaphylaxis, in which a dramatic increase in serum tryptase levels can be attained (31). Further, tryptase is considered as a potential biomarker for a range of additional pathologies, including asthma (8) and melanoma (32). On a different angle, it has been found that certain individuals harbor an increased copy number of the tryptase alpha gene, which leads to elevated basal tryptase levels, associated with a complex pathology denoted hereditary alpha tryptasemia syndrome (33).

Altogether, tryptase is thus implicated as a diagnostic tool in a wide range of disorders. However, beyond its diagnostic value, there is more limited insight as to whether tryptase has a functional impact on any of these settings. In this study we provide novel insight into this issue, by examining the functional impact of tryptase on mast cell leukemia cells. Our findings reveal that tryptase has a profound effect on the processing of nuclear core histones, in the context of cell death induced in response to a variety of triggers. Notably, the extent of histone processing was most profound in cells where cell death was triggered by exposure to LLME. LLME is a lysosomotropic agent that has the capacity to induce mast cell secretory granule permeabilization. This will lead to the release of tryptase from the granules into the cytosol, and it would thus be expected that cell death in response to such a mechanism would lead to more evident effects on histone processing vs. triggering of cell death by other mechanisms (which may not cause granule permeabilization to the same extent). It was also notable that, out of the various core histones, H3 was most susceptible, being degraded in response into each of the adopted cell death-triggering agents. This probably reflects that H3 has a more extended N-terminal tail than the other core histones, protruding outside of the nucleosomes. Most likely, the N-terminal tail of H3 is thereby highly susceptible to the proteolytic action of tryptase. Moreover, since the H3 N-terminal tail is structurally flexible, it is likely that it can enter the central pore of tryptase without major steric hindrance.

We also noted that cell death induced by LLME was accompanied by a reduction in several epigenetic marks deposited on H3, including H3K9me2 and H3K4me1. H3K9me2 is strongly associated with transcriptional repression (34), whereas H3K4me1 is enriched at active enhancers (35). The reduction of these marks in HMC-1 cells exposed to cytotoxic agents may thus reflect effects on regulation of the transcriptional program. Intriguingly, the reduction in the levels of these marks was completely abolished in cells treated with tryptase inhibitor, introducing the notion that tryptase can regulate transcriptional events mediated through these epigenetic marks. These marks are found close to the N-terminus of H3, and a likely explanation for the observed effects would be that tryptase executes proteolytical cleavage in the N-terminal tail of H3, such that these marks are erased.

In addition to influencing H3K9me2 and H3K4me1, our data also indicate that tryptase has the capacity to remove H3S10p and H2BK16ac marks in response to triggering of cell death. The H3S10p mark is known to have a role in the regulation of cell apoptosis (28), and our data thus suggest that tryptase has the capacity to modulate apoptotic responses mediated by this epigenetic mark. In line with this, we noted that tryptase inhibition has a strong impact on the mechanism of cell death, with tryptase inhibition causing a diversion of the cell death mode from apoptosis to late apoptosis/necrosis. However, further investigations are needed to determine if this diversion is a direct effect of the altered epigenetic landscape induced by proteolytic effects of tryptase on core histone tails.

An interesting observation was that tryptase inhibition caused an increase in the levels of certain histone marks (H3K4me1 and H3K9me2) even at baseline conditions, i.e., in the absence of cell death-triggering agent. This implies that tryptase may have an impact in the nuclear compartment even in viable cells. In line with this, we found here that tryptase, in addition to its location within secretory granules, also is found within the nuclear compartment of the human mast cell leukemia cells, and there is also previous evidence that tryptase can be found in the nucleus of non-transformed mast cells (36, 37). Hence, tryptase can impact on core histones not only during cell death, when large amounts of tryptase are released from granules, but also at steady state. Indeed, our data show that tryptase inhibition caused an increased expansion of the HMC-1 cultures under baseline conditions, and that this was attributed to an enhanced rate of proliferation. Potentially, this increase in proliferation could be due to tryptase-mediated effects on the epigenetic landscape in the HMC-1 nucleus, such as our observed increase in the H3K4me1 and H3K9me2 marks in HMC-1 cells treated with tryptase inhibitor. However, further investigations will be needed to establish the causative relationship between tryptase inhibition and regulation of cellular proliferation.

## Materials and Methods

### Reagents

UNC-0638 (cat no U4885) was from Sigma-Aldrich (Steinheim, Germany), UNC-1999 (cat no S7165) from Selleckchem (Houston, TX), H-Leu-Leu-OMe (LLME) (cat no 4000725.0005) from Bachem (Bubendorf, Switzerland). Nafamostat mesylate (cat no N0289), staurosporine (cat no S4400), mefloquine (cat no M2319), Pefabloc SC (cat no 11429868001), pepstatin A (cat no 516481) and E-64d (cat no E8640) were from Sigma-Aldrich (Steinheim, Germany). Rabbit anti-H3K9me2 antibody (cat no 07-441) was from EMD-Millipore (Darmstadt, Germany). Rabbit anti-H3K4me1 (cat no 5323S) monoclonal antibody was from Cell Signaling Technology (Danvers, MA). Rabbit anti-histone 2A (H2A) (cat no ab177308), H2B (cat no ab1790), H3 (cat no ab1791) and H4 (cat no ab177840) antibodies, mouse anti-H3K27me3 (cat no ab6002) monoclonal antibody and rabbit anti-H3S10p (cat no ab272166) polyclonal antibody were from Abcam (Cambridge, UK). A mouse monoclonal antibody to β-actin (cat no sc-517582) was from Santa Cruz Biotechnology (Santa Cruz, CA). Rabbit anti-H2BS14p polyclonal antibody (cat no PA5-105775) was from Invitrogen (Eugene, OR). Rabbit anti-H2BK16ac (cat no 39121) was from Active Motif (Carlsbad, CA).

### Culture of Mast Cell Leukemia Cells

The human mast cell line (HMC-1) was cultured in Iscove’s Modified Dulbecco’s medium (IMDM) (cat no 11504556; Invitrogen, Carlsbad, CA) supplemented with 10% heat-inactivated fetal bovine serum (FBS) (cat no 11573397; Invitrogen, Carlsbad, CA), 2 mM L-glutamine (cat no 59202C; Sigma Aldrich), 100 μg/mL streptomycin (Sigma Aldrich), 100 IU/mL penicillin (Sigma Aldrich; cat no P4333) and 1.2 mM 1-thioglycerol (cat no M6145; Sigma Aldrich). The medium was replaced every third day; cells were cultured at 0.3 x 10^6^ cells/ml at 37°C with 5% CO_2_ and used for at most 10 passages after initial thawing.

### Cell Viability Assessment

To evaluate the effects of different inhibitors on cell viability, cells were preincubated with or without inhibitors. The Cell Titer-Blue cell viability assay (cat no G8080; Promega, Carlsbad, CA) was used to assess cytotoxicity. Triplicates of 0.5 x 10^6^ cells/ml were resuspended in complete culture medium, transferred into a 24-well flat-bottomed plates and incubated overnight at 37°C with 5% CO_2_. The next day, cells were either treated with inhibitors or left non-treated and incubated at 37°C with 5% CO_2_. After 24 h incubation, 90 μl of the cell suspensions were transferred into 96-well flat-bottomed plates, followed by adding 10 μl of cell viability reagent, and incubation for 1 h at 37°C (5% CO_2_) followed by measuring of fluorescence using a microplate reader (M200-Infinite; Tecan, Männedorf, Switzerland) at 560 nm for excitation and 590 nm for emission.

### Flow Cytometry Assessment of Cell Death

Cells were treated with UNC-0638 (25 μM), UNC-1999 (50 μM), LLME (400 μM), staurosporine (1 μM) or left untreated prior to incubation for 24h (at 37°C, 5% CO_2_). Subsequently, cells were centrifuged (400 x g, 5 min), washed with cold PBS and cell death was assessed by staining with Annexin V-FITC (BD Biosciences, Franklin Lakes, NJ) and Draq7™ (Biostatus, Shepshed, UK) diluted in Annexin V binding buffer (BD Biosciences). Cells were analyzed with a BD Accuri™ C6 Plus Flow Cytometer (BD Biosciences), and the FlowJo software (BD Biosciences) was used for data analysis. Cells were counted using a Countess II FL automated cell counter (Thermo Fisher Scientific, Waltham, MA). Protease inhibitors (Pefabloc SC, Nafamostat, E-64d, EDTA and pepstatin A) were preincubated for 30 min before adding the UNC-0638, UNC-1999, LLME or Staurosporine.

### Western Blot

Western blot analysis was performed as previously described (38). Equal amounts of cells were diluted in 100 μl of Laemmli sample buffer and boiled for 10 min at 95°C in a heating-block. Thereafter, samples were separated by SDS-PAGE (Novex WedgeWell 4-20% Tris-Glycine Gels, Thermo Fisher Scientific). Gels were transferred onto Immobilon-FL PVDF membranes (EMD-Millipore), followed by blocking in Odyssey Blocking buffer (LI-COR, Lincoln, NE) mixed with an equal volume of TBS or PBS, prior to overnight-incubation at 4°C with one of the following primary antibodies (1:1000; in blocking buffer): anti-histone 2A (H2A), H2B, H4, H3K4me, H3K9me3, H3K27me3, H2B-S14p or H3-S10p antibody. Thereafter, membranes were washed (3 × 10 min) with PBS or TBS/0.1% Tween-20, followed by a final wash (10 min) with PBS or TBS and then by incubation with secondary fluorescent antibodies (1:15,000; in TBS or PBS). The secondary antibodies were from LI-COR (IRDye 800 CW Donkey Anti-Rabbit IgG, IRDye 680 CW Donkey Anti-Mouse IgG, and IRDye 800 Donkey; LI-COR). After one hour of incubation at room temperature, the membranes were washed and scanned by using the Odyssey CLx Imaging System (LI-COR). Western blots were quantified and analyzed using Image Studio Lite Software (LI-COR). The targeted proteins were normalized to the loading control (β-actin).

### Confocal Microscopy

Aliquots of 200 μl with 0.3 x 10^6^ cells were dropped into round areas on microscopic glasses made with liquid-repellent pen. Suspensions were kept for 15 min and liquid was removed carefully with filter paper. The remaining cells were left to dry for 15 min. Samples were fixed and permeabilized with methanol for 10 min, followed by a 15 min drying step. On the top of each area, 100 μl of the mouse monoclonal human mast cell tryptase antibody (Abcam, Cambridge, UK) (1:500) in Tris-buffered saline (TBS)/1% bovine serum albumin (BSA) and/or isotype control at the same concentration were left overnight at 4°C, followed by 3 x washing with TBS-T. 100 μl of anti-mouse Alexa-488 conjugated antibody in TBS/1% BSA was added to each slide. The slides were kept at room temperature in the dark for 2 h and then washed three x with TBS-T. NucBlue (Life Technologies, Carlsbad, CA) was added, followed by 3 x washing with TBS-T. The slides were mounted with SlowFade™ diamond antifade mounting medium (Invitrogen, Eugene, OR) and cover glasses. Samples were analyzed using a laser-scanning microscope equipped with ZEN 2009 software (LSM 710, Carl Zeiss, Berlin, Germany).

### Cell Growth in the Presence of Nafamostat

HMC-1 cells were initially diluted to 0.1 x 10^6^ cells/ml and 1 ml cell suspensions were distributed in triplicates into 24-well plates. Nafamostatat mesylate was added at different concentrations and cells were incubated at 37°C with 5% CO_2_ for different time periods. Cell viability was accessed using Annexin-V and Draq7 (Biostatus, Shepshed, UK) 24 h after nafamostat treatment to evaluate toxicity. Samples were analyzed by flow cytometry using a BD Accuri C6 plus flow cytometer (BD Biosciences). Cells left under culture conditions were subcultured for 4 and 7 days. Cell counts, viability and size were measured using the Countess II FL automated cells counter (Life Technologies).

### EdU Labeling

1 ml of 0.1 x 10^6^ cells/ml were distributed into 24-well plates. Cells were treated with 10 μM nafamostat and kept at 37°C in 5% CO_2_ for 48 h. Two hours before harvesting the cells, 10 μM EdU was added. Cells were then stained using the Click-iT™ EdU Alexa 488 flow cytometry kit (Life Technologies). Flow cytometry analysis was performed (BD Accuri C6 plus). Data from 10,000 events/sample were collected and analyzed by the Flow Jo software (Ashland, OR).

### Statistical Analysis

Statistically significant differences between groups were determined by using one-way ANOVA with *post hoc* Dunnett’s multiple comparison test. All graphs were prepared, and statistics calculated using GraphPad Prism 8.0 (GraphPad software Inc., San Diego, CA). A *P*-value of less than 0.05 was considered significant.

## Data Availability Statement

The original contributions presented in the study are included in the article/**Supplementary Material**. Further inquiries can be directed to the corresponding authors.

## Author Contributions

SA designed and performed experiments, and wrote the paper. FR designed, supervised and performed experiments, interpreted data and contributed to the writing of the paper. GP designed and planned the study, and wrote the paper. All authors contributed to the article and approved the submitted version.

## Funding

This study was supported by grants from The Swedish Research Council, The Swedish Heart and Lung Foundation, The Swedish Research Council, The Swedish Cancer Foundation, Knut & Alice Wallenberg Foundation and the Saudi Arabian Ministry of Education.

## Conflict of Interest

The authors declare that the research was conducted in the absence of any commercial or financial relationships that could be construed as a potential conflict of interest.

## Publisher’s Note

All claims expressed in this article are solely those of the authors and do not necessarily represent those of their affiliated organizations, or those of the publisher, the editors and the reviewers. Any product that may be evaluated in this article, or claim that may be made by its manufacturer, is not guaranteed or endorsed by the publisher.

## Abbreviations

MC, mast cell; LLME, H-Leu-Leu-OMe; H, histone.
