# Supplementary material for: Tryptase Regulates the Epigenetic Modification of Core Histones in Mast Cell Leukemia Cells
Source: Front Immunol. 2021 Dec 2;12:804408. doi: 10.3389/fimmu.2021.804408 (PMC8674432; doi:10.3389/fimmu.2021.804408)
Supplement: Supplementary file 1 [file DataSheet_1.pdf]

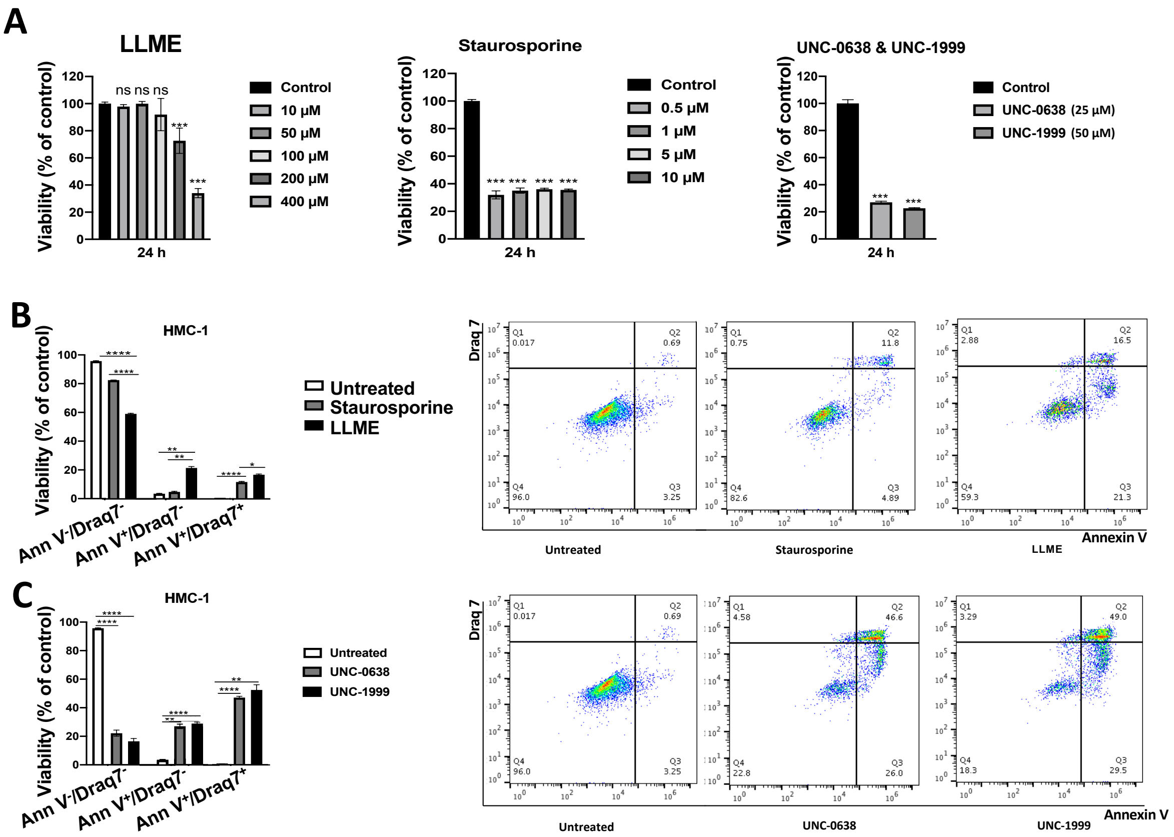

Suppl. Figure 1. H-Leu-Leu-OMe (LLME), staurosporine and histone methyltransferase inhibitors reduce the viability of HMC-1 mast cell leukemia cells. (A) HMC-1 cells (0.5 x 10^6 cells/ml) were either left untreated or treated with LLME, Staurosporine, UNC-0636 (histone methyltransferase inhibitor) or UNC-1999 (histone methyltransferase inhibitor) at the indicated concentrations and time periods. Cell viability was then quantified using the CellTiter-Blue Cell Viability Assay. (B) HMC-1 subjected to LLME, Staurosporine, UNC-0636 or UNC-1999 for 24 hours were analyzed by flow cytometry for Annexin V/Draq7 positivity. Data are representative of at least two independent experiments and are given as mean values ±SEM (n = 3). * $p \leq 0.05$; ** $p \leq 0.01$; *** $p \leq 0.001$; **** $p \leq 0.0001$
